# Bone Turnover and Metabolism in Patients with Early Multiple Sclerosis and Prevalent Bone Mass Deficit: A Population-Based Case-Control Study

**DOI:** 10.1371/journal.pone.0045703

**Published:** 2012-09-19

**Authors:** Stine Marit Moen, Elisabeth Gulowsen Celius, Leiv Sandvik, Magritt Brustad, Lars Nordsletten, Erik Fink Eriksen, Trygve Holmøy

**Affiliations:** 1 Department of Neurology, Oslo University Hospital Ullevål, Oslo, Norway; 2 Section of Epidemiology and Biostatistics, Oslo University Hospital Ullevål, Oslo, Norway; 3 Department of Community Medicine, University of Tromsø, Tromsø, Norway; 4 Orthopedic Department, Oslo University Hospital Ullevål, Oslo, Norway; 5 Department of Endocrinology, Oslo University Hospital, Oslo, Norway; 6 Department of Neurology, Akershus University Hospital, Lørenskog, Norway; 7 Institute of Clinical Medicine, University of Oslo, Oslo, Norway; University Hospital La Paz, Spain

## Abstract

**Background:**

Low bone mass is prevalent in ambulatory multiple sclerosis (MS) patients even shortly after clinical onset. The mechanism is not known, but could involve shared etiological risk factors between MS and low bone mass such as hypovitaminosis D operating before disease onset, or increased bone loss after disease onset. The aim of this study was to explore the mechanism of the low bone mass in early-stage MS patients.

**Methodology/Principal Findings:**

We performed a population-based case-control study comparing bone turnover (cross-linked N-terminal telopeptide of type 1 collagen; NTX, bone alkaline phosphatase; bALP), metabolism (25-hydroxy- and 1, 25-dihydroxyvitamin D, calcium, phosphate, and parathyroid hormone), and relevant lifestyle factors in 99 patients newly diagnosed with clinically isolated syndrome (CIS) or MS, and in 159 age, sex, and ethnicity matched controls. After adjustment for possible confounders, there were no significant differences in NTX (mean 3.3; 95% CI −6.9, 13.5; p = 0.519), bALP (mean 1.6; 95% CI −0.2, 3.5; p = 0.081), or in any of the parameters related to bone metabolism in patients compared to controls. The markers of bone turnover and metabolism were not significantly correlated with bone mass density, or associated with the presence of osteoporosis or osteopenia within or between the patient and control groups. Intake of vitamin D and calcium, reported UV exposure, and physical activity did not differ significantly.

**Conclusions/Significance:**

Bone turnover and metabolism did not differ significantly in CIS and MS patients with prevalent low bone mass compared to controls. These findings indicate that the bone deficit in patients newly diagnosed with MS and CIS is not caused by recent acceleration of bone loss, and are compatible with shared etiological factors between MS and low bone mass.

## Introduction

Multiple sclerosis (MS) is a demyelinating inflammatory disease of the central nervous system [1]. Patients with long-standing MS have an increased risk of osteoporosis and fractures due to reduced bone mass and falls [2]–[5]. As in many other chronic diseases, disability leading to disuse and reduced mechanical loading of bone is likely an important risk factor for osteoporosis in patients with long-standing MS [6], [7]. The roles of other possible factors contributing to reduced bone mass or bone mineral density (BMD), such as low levels of vitamin D, medications in the course of disease, and the inflammatory process itself, are less clear [8].

MS results from an interplay between genetic susceptibility and environment, and growing evidence suggests that hypovitaminosis D is a risk factor [9]. Vitamin D is essential for bone growth, preservation, and mineral homeostasis, and has also immunomodulatory effects [10]. Environmental factors such as hypovitaminosis D acting from conception to early adult life might be of importance for the risk of MS [11]–[13]. During the same period the skeleton acquires a peak bone mass influenced by genetic and environmental factors, including vitamin D status [14]. If hypovitaminosis D is a risk factor for MS, it is conceivable that MS patients have low bone mass already at the time of diagnosis. We have earlier found that low bone mass was more prevalent in newly diagnosed patients with MS and clinically isolated syndrome (CIS) suggestive of demyelinating disease than in controls [15]. This finding is compatible with the hypothesis that MS and osteoporosis share etiological factors, and that the bone deficit in the newly diagnosed patients could be explained by low bone mass before disease onset. An alternative explanation could be increased bone loss after disease onset, which would be reflected by increased bone turnover or perturbated bone metabolism at the time of BMD assessment.

The bone tissue is continually adapting to various inputs, and coupled bone formation and bone resorption is a dynamic process that occurs throughout life [16]. While BMD reflects the sum of peak bone mass and the amount of subsequent bone loss, changes in bone turnover markers occur more rapidly and provide a dynamic view into the current bone metabolism. The bone resorption marker cross-linked N-terminal telopeptide of type 1 collagen (NTX) is a breakdown product of type 1 collagen and reflects degradation of bone matrix, whereas bone alkaline phosphatase (bALP) secreted from osteoblasts reflects bone formation [17]. The overall aim of this study was to explore the mechanism of the low bone mass previously recorded in patients newly diagnosed with CIS and MS compared to controls [15]. We therefore assessed NTX, bALP, vitamin D metabolites, and other biochemical and lifestyle factors related to bone metabolism in patients newly diagnosed with MS or CIS and controls.

## Methods

### Ethics Statement

The study was approved by the Regional Ethics Committee for Medical Research in South-Eastern Norway Regional Health Authority, the Review Board of Oslo University Hospital Ullevål, and the National Population Registry, Norway and carried out in accordance with the Helsinki Declaration. Written informed consent was obtained from all participants.

### Study Design and Participants

The design of this population-based case-control study has been described previously [15]. Briefly, all 107 patients living in Oslo and diagnosed with CIS or MS according to the McDonald criteria [18] between January 2005 and January 2008 were invited to participate. Ninety-nine (92.5%) patients participated in the study. The mean duration from first symptom of demyelinating disease to study examination was 1.6±1.3 years, and mean expanded disability status scale (EDSS) score was 1.4±1.1. Age-, sex-, and ethnicity-matched controls were randomly selected from the National Population Registry, and 275 (45.8%) of the subjects selected responded, of whom 219 (79.6%) were willing to participate. Of these, one control was randomly selected for each patient. In addition, the patients recruited 60 unrelated controls of the same sex, age (±5 years), ethnicity, and for women the same menopausal phase.

### Data Collection

Blood and urine samples from paired patients and controls were preferentially collected at the same date or during a restricted time period (±2 weeks) from May 2007 to September 2008. Serum 25-hydroxyvitamin D (25(OH)D) and 1,25-dihydroxyvitamin D (1,25(OH)_2_D) were measured by competitive radioimmunoassay (RIA) (DiaSorin, Stillwater, MN, USA), vitamin D binding protein (DBP) was measured with RIA using human Gc-globulin (Sigma, St.Louis, USA) and rabbit polyclonal anti-Gc-globulin antibody (DakoCytomation, Glostrup, Denmark), and parathyroid hormone (PTH) was measured with non-competitive immunoluminometric assay (ILMA, Immulite 2500 Diagnostic Products Corporation, Los Angeles, Ca, USA) at the Hormone Laboratory at Oslo University Hospital Aker. The RIA detected both the D_2_ and D_3_ forms of 25(OH)D and 1,25(OH)_2_D. The coefficients of variation (CV) were 6% intra-assay and 13–16% inter-assay for 25-(OH)D, 8–12% intra-assay and 14–20% inter-assay for 1,25(OH)_2_ D, 5% intra-assay and 12–14% inter-assay for DBP, 3–5% intra-assay and 8–9% inter-assay for PTH. Serum bALP was measured by an enzyme immunoassay kit (Metra Biosystems Inc., Mountain View, CA, USA), and the enzyme activity was measured in U/l where one unit of bALP activity was defined as 1 µmol of p-nitrophenyl phosphate hydrolyzed per minute at 25°C in 2-amino-2 methyl-1-propanol. The intra-assay and inter-assay CV values were 2–6% and 12–13%, respectively. Serum ionized calcium (Ca), creatinine, and phosphate were measured according to standard laboratory techniques. NTX in the second morning void urine was measured by competitive enzyme immunoassay (12–20% intra-assay and 12–19% inter-assay CV).

A questionnaire including medication, comorbidity, menstrual status, smoking, alcohol, diet (food frequency), physical activity, and sun exposure was mailed to the participants and collected at the time of study examination. Comorbidity was assessed by asking the participants if they had any chronic diseases with the answer categories “yes” or “no”, with further specification if “yes”. Relevant medications with possible skeletal effects were noted to determine possible confounding [15]. A validated self-administered food frequency questionnaire [19] was used to assess average intake of vitamin D (µg per day) and calcium (mg per day) the preceding year. The nutrients were computed using nutrient values from the Norwegian Food Composition Table (2006) and analyzed with Statistical Analysis System (SAS) software package, version 9.1 (SAS Institute Inc, Cary, NC) at the Department of Community Medicine, University of Tromsø, Norway. Current physical activity was recorded on a scale ranging from 1 to 10 and categorized as “low” (1–3), “moderate” (4–7), and “high” (8–10) [20]. The questionnaire also covered ultraviolet (UV)/sun exposure including solarium use, sun holidays in southern latitudes (Mediterranean and near equator), and sun tanning in Norway and in northern latitudes the two months prior to blood and urine sampling [19]. The level of disability (EDSS) [21] and other details related to past medical history, including recent childbirth and breastfeeding (last 12 months), were recorded by a neurologist.

### Missing Data

Four PTH, three Ca, one bALP, one phosphate, and three NTX values from controls, and one phosphate value from a patient were missing due to technical errors. One patient and six controls did not answer the question about current physical activity. One patient and one control did not answer the question about solarium use, and one patient did not answer the question about sun tanning in Norway the preceding two months.

### Statistical Analysis

Statistical analyses included independent-samples two-tailed t tests for continuous variables, Mann-Whitney U test for not normally distributed data, and Chi-square test for dichotomous variables for comparison of groups. Unpaired analyzes were used because the parity of the matching was incomplete. Results are expressed as mean ± SD or as number (percentages) unless otherwise stated. Pearson’s or Spearman’s rank correlation coefficients (r) were calculated for bivariate correlations between variables depending on their distribution. Possible confounders were identified by checking whether the variable in question was associated with the measure, or whether it differed significantly between patients and controls. Adjustments for the possible confounders were performed using multivariate linear regression for numerical outcomes. The possible confounders were analyzed as independent variables separately and simultaneously in the regression models. The following outcome variables were analyzed as dependent: NTX, phosphate, left femoral neck BMD, total body BMD, and nondominant radius BMD. Multiple linear regression was also used to analyze the interactions between bone mass status (osteoporosis/osteopenia or normal) and participant status (patient or control) in the association analyses of biochemical parameters. The interaction term was a combination of bone mass status (osteoporosis or osteopenia/normal bone mass) and participant status (patient/control). A variance inflation factor >5 was used as criterion for multicollinearity. Missing values were not replaced. Statistical significance was defined as p<0.05. All analyses were conducted using SPSS (version 18, SPSS, Chicago, IL).

## Results

The blood and urine samples from paired patients and controls were obtained within mean 4.4±8.7 days (median 0, range 0–64.0). Patients exhibited higher urine NTX and serum phosphate levels compared to controls (Table 1). The other biochemical parameters of bone turnover and metabolism, including vitamin D status–serum levels of 25(OH)D, 1, 25(OH)_2_D, DBP, bALP, Ca, and PTH–did not differ between patients and controls. Two (2.0%) patients and 3 (1.9%) controls had vitamin D deficiency defined as 25(OH)D <25 nmol/l, and 21 (21.2%) patients and 31 (19.5%) controls were below 50 nmol/l. Thirty-eight (38.4%) patients and 60 (37.7%) controls had 25(OH)D ≥75 nmol/l.

**Table 1:** Biochemical markers (mean ± SD) of vitamin D status, bone turnover and metabolism in patients and controls.

	Patients (n = 99)	Controls (n = 159)	95% CI	p value
**25(OH)D** (nmol/l)	68.4±24.5	68.5±22.9	−6.0, 5.9	0.992
**1,25(OH)_2_D** (pmol/l)	119.9±43.7	127.0±44.9	−18.4, 4.1	0.211
**PTH** (pmol/l)	3.55±1.89	3.68±1.83	−0.60, 0.34	0.585
**Ionized calcium** (mmol/l)	1.27±0.03	1.27±0.04	−0.01, 0.01	0.986
**Phosphate** (mmol/l)	1.14±0.15	1.09±0.16	0.01, 0.09	0.012
**DBP** (µmol/l)	4.28±0.72	4.18±0.73	−0.08, 0.29	0.255
**Creatinine** (µmol/l)	66.2±11.1	69.0±11.8	−5.7, 0.1	0.060
**bALP** (U^a^/l)	24.2±7.6	22.6±7.2	−0.2, 3.5	0.081
**NTX** (nmol/l BCE^b^/mmol/l Cr^c^)	54.7±42.2	44.6±23.0	2.1, 18.2	0.030

25(OH)D: 25-hydroxyvitamin D, 1,25(OH)_2_D: 1,25-dihydroxyvitamin D, PTH: parathyroid hormone, DBP: vitamin D binding protein, bALP: bone alkaline phosphatase, NTX: cross-linked N-terminal telopeptide of type 1 collagen (all measured in serum, except NTX that was measured in urine).

a Enzyme activity measured in U/l, 1U: one unit of bALP activity was defined as 1 µmol of p-nitrophenyl phosphate hydrolyzed per minute at 25°C,

b BCE: Bone Collagen Equivalents,

c Cr: Creatinine. Comparison of groups was calculated using independent two-tailed t test. CI: mean difference confidence interval.

In order to identify possible confounders we assessed factors that may influence bone metabolism. Use of systemic glucocorticoids, current smoking, use of alcohol, recent childbirth, and breastfeeding differed significantly between patients and controls (Table 2). There was no significant difference in estimated daily intake of vitamin D and calcium between patients and controls, or in current physical activity, use of solarium, or sun tanning in Norway or at lower latitudes in the preceding two months. Participants who had recently given birth, were breastfeeding, had begun menopausal transition, or reported no sun exposure in northern latitudes the preceding 2 months revealed higher NTX levels than those not being exposed to these factors (p<0.001, p<0.001, p = 0.043, and p = 0.001, respectively). NTX did not differ significantly between those who had or had not been exposed to the other variables that could affect bone metabolism or remodeling listed in Table 2.

**Table 2:** Distribution of factors that may influence current bone metabolism.

	Patients (n = 99)	Controls (n = 159)	p value
**Vitamin D intake** (µg per day), **preceding year**, (mean ± SD)	8.1±8.1	8.0±6.4	0.899
**Calcium intake intake** (mg per day), **preceding year**, (mean ± SD)	648±293	633±268	0.667
**UV/sun exposure preceding 2 months**, n (%)			
Solarium >1 per month	15 (15.3)	23 (14.6)	0.870
Sun tanning in northern latitudes ≥1 week	17 (17.3)	35 (22.0)	0.366
Sun tanning in southern latitudes^a^ ≥1 week	10 (10.1)	21 (13.2)	0.455
**Current physical activity**, n (%)			0.057
Low	23 (23.5)	19 (12.4)	n/a
Moderate	61 (62.2)	114 (74.5)	n/a
High	14 (14.3)	20 (13.1)	n/a
**Ever used iv or po glucocorticoids**, n (%)	38 (38.4)	1 (0.6)	<0.001
**Ever used inhalation glucocorticoids**, n (%)	3 (3.0)	7 (4.4)	0.579
**Have other relevant diseases**, n (%)	8 (8.1)	11 (6.9)	0.728
**Use other relevant medication**, n (%)	8 (8.1)	10 (6.3)	0.583
**Current smokers**, n (%)	31 (31.3)	27 (17.0)	0.007
**Alcohol more often than 1/week**, n (%)	33 (33.3)	76 (47.8)	0.022
**Begun menopausal transition** b, n (%)	8 (11.3)	10 (8.5)	0.539
**Absent menstruation** b, n (%)	15 (21.1)	15 (12.8)	0.132
**Hormonal treatment** b, n (%)	11 (15.5)	15 (12.8)	0.607
**Recently given birth** b, n (%)	7 (9.9)	2 (1.7)	0.011
**Breastfeeding** b, n (%)	6 (8.5)	1 (0.9)	0.008

iv: intravenous, po: per oral.

a Mediterranean or other destinations nearer equator than Norway.

b percentages of female participants (female patients, n = 71; female controls, n = 117). Comparison of groups was calculated using Chi-square test and independent-samples two-tailed t test.

The impact on NTX of potential confounders (variables that either differed significantly between patients and controls or were significantly associated with NTX) were analyzed both separately and simultaneously with multiple linear regression. NTX was analyzed as a dependent variable and the following as independent variables: use of systemic glucocorticoids, current smoking, use of alcohol, recent childbirth, breastfeeding, begun menopausal transition, sun tanning in northern latitudes preceding two months, and phosphate. After adjusting for these factors, the difference in NTX between patients and controls was no longer significant (Table 3).

**Table 3:** Differences in urine NTX between patients and controls, without and with adjustment for possible confounders.

	β (95% CI)	p value
**Unadjusted**	10.2 (2.1, 18.2)	0.014
**Adjusted for:**		
Recent childbirth	6.9 (−0.8, 14.7)	0.079
Breastfeeding	6.0 (−1.5, 13.6)	0.117
Ever used iv or po glucocorticoids	8.1 (−1.3, 17.5)	0.090
Current smoking	10.6 (2.4, 18.8)	0.011
Alcohol use	9.7 (1.6, 17.9)	0.020
Begun menopausal transition	9.6 (0.3, 18.9)	0.044
Sun tanning in northern latitudes preceding 2 months	9.6 (1.6, 17.7)	0.019
Phosphate	8.9 (0.7, 17.0)	0.033
Recent childbirth, systemic glucocorticoids, currentsmoking, alcohol use, begun menopausal transition, sun tanning innorthern latitudes preceding 2 months, and phosphate.	3.6 (−6.9, 14.1)	0.497
Breastfeeding, systemic glucocorticoids, current smoking,alcohol use, begun menopausal transition, sun tanning innorthern latitudes preceding 2 months, and phosphate.	3.3 (−6.9, 13.5)	0.519

β: unstandardized β coefficient, CI: β confidence interval. NTX (cross-linked N-terminal telopeptide of type 1 collagen) was analyzed as dependent and the following as independent variables (separately and simultaneously): recent childbirth, breastfeeding, systemic glucocorticoids, current smoking, alcohol use, began menopausal transition, sun tanning in northern latitudes, and phosphate. Recent childbirth and breastfeeding were not included simultaneously in the regression analysis due to their high correlation (r = 0.921; p<0.001).

As patients exhibited higher serum levels of phosphate than controls, the impact of possible confounders on phosphate was investigated. Phosphate was higher in participants who had recently given birth (p<0.001), were breastfeeding (p<0.001), or had used systemic glucocorticoids (p = 0.017) compared to those who had not been exposed to these factors. Phosphate was included as a dependent variable and use of systemic glucocorticoids, recent childbirth, and breastfeeding as independent variables in the regression model (again recent childbirth and breastfeeding were not included simultaneously in the regression analysis due to the high correlation).The difference in phosphate between patients and controls did not remain significant when adjusting for recent childbirth and use of systemic glucocorticoids (β = 0.026; 95% CI −0.019, 0.071; p = 0.262), or breastfeeding and use of systemic glucocorticoids (β = 0.025; 95% CI −0.020, 0.070; p = 0.273).

In order to further examine whether there were perturbations in bone metabolism in the CIS and MS patients, we compared the correlations between markers of bone turnover and metabolism between patients and controls. Both bALP and NTX showed positive correlations in both patients (r = 0.553; p<0.0001) and controls (r = 0.405; p<0.0001). Serum 25(OH)D and 1,25(OH)_2_D also exhibited positive correlations in patients (r = 0.342; p = 0.001) and in controls (r = 0.255; p = 0.001). There was a significant inverse correlation between 25(OH)D and PTH in the controls (r = −0.218; p = 0.007), but this inverse correlation was not significant among the patients (r = −0.123; p = 0.226). There was a significant inverse correlation between PTH and Ca in both patients (r = −0.280; p = 0.005) and controls (r = −0.402; p<0.001). The strength of these correlations did not differ between patients and controls.

There were no significant differences in the levels of 25(OH)D, 1,25(OH)_2_D, bALP, NTX, PTH, or Ca among patients or controls with either osteoporosis or osteopenia compared to those with normal bone mass (Table 4). Applying multiple linear regression with a combination of bone mass status (osteoporosis or osteopenia/normal bone mass) and participant status (patient/control) as interaction term, we found that the associations between the biochemical parameters and bone status were unaffected by participant status (data not shown).

**Table 4:** Biochemical measures (mean ± SD) of vitamin D status, bone turnover and metabolism in patients and controls with different bone status.

	Patients				Controls			
	Low BM(n = 50)	Normal BM(n = 49)	Mean diff (95% CI)	p value	Low BM(n = 59)	NormalBM(n = 100)	Mean diff (95% CI)	p value
**25(OH)D**	67.1±23.7	69.8±25.5	−2.6 (−12.4, 7.2)	0.598	66.9±22.3	69.4±23.2	−2.5 (−9.9, 4.9)	0.513
**1,25(OH)_2_D**	123.2±46.0	116.5±41.4	6.6 (−10.8, 24.1)	0.452	127.9±39.9	126.5±47.8	1.4 (−13.3, 16.0)	0.855
**PTH**	3.64±1.91	3.46±1.89	0.18 (−0.58, 0.95)	0.637	3.90±1.92	3.54±1.78	0.36 (−0.24, 0.96)	0.238
**ionCa**	1.27±0.04	1.27±0.03	0.003 (−0.01, 0.02)	0.648	1.27±0.04	1.28±0.04	−0.009 (−0.02, 0.003)	0.135
**bALP**	24.6±8.9	23.9±6.1	0.7 (−3.8, 2.4)	0.648	22.3±6.7	22.7±7.5	−0.4 (−2.0, 2.7)	0.764
**NTX**	60.9±52.8	48.5±26.6	12.5 (−29.2, 4.3)	0.142	48.8±25.0	42.1±21.6	6.7 (−14.2, 0.8)	0.080

Low BM: low bone mass; osteoporosis or osteopenia, mean diff: mean difference, 25(OH)D: 25-hydroxyvitamin D, 1,25(OH)_2_D: 1,25-dihydroxyvitamin D, PTH: parathyroid hormone, ionCa: ionized calcium, bALP: bone alkaline phosphatase, NTX: cross-linked N-terminal telopeptide of type 1 collagen. Comparison of groups was calculated using independent two-tailed t test. CI: confidence interval.

There was no significant correlation between 25(OH)D and BMD at any of the skeletal sites in patients or controls (Table 5). There was, however, a significant inverse correlation between 1,25(OH)_2_D and BMD in the left femoral neck, total body, and nondominant ultradistal radius among the patients, but not among the controls (Table 5). The correlations between BMD and 1,25(OH)_2_D did not remain significant after adjusting for variables associated with BMD at each skeletal site (i.e. use of inhalation glucocorticoids) or variables which differed significantly between patients and controls (i.e. use of systemic glucocorticoids, current smoking, use of alcohol, recent childbirth, breastfeeding, NTX, and phosphate) in multivariate linear regression analyses (left femoral neck; p = 0.518, total body; p = 0.586, and nondominant ultradistal radius; p = 0.071), using BMD at each skeletal site as dependent variables and use of inhalation glucocorticoids, use of systemic glucocorticoids, current smoking, use of alcohol, recent childbirth, breastfeeding, NTX, and phosphate as independent variables. Finally, there were no significant associations (analyzed with linear regression) between EDSS and any of the markers of bone turnover or metabolism in the patient group (data not shown).

**Table 5:** Correlations between bone mineral density (BMD) and vitamin D metabolites and PTH.

	Patients (n = 99)		Controls (n = 159)	
	Pearson’s r	p value	Pearson’s r	p value
**Correlation between 25(OH)D and BMD**				
Lumbar spine	0.008	0.939	0.108	0.176
Total hip	0.130	0.198	0.046	0.570
Left femoral neck	0.107	0.291	0.046	0.565
Right femoral neck	0.094	0.354	0.048	0.547
Total body	0.083	0.414	−0.024	0.767
Nondominant ultradistal radius	−0.063	0.536	−0.027	0.741
**Correlation between 1,25(OH)_2_D and BMD**				
Lumbar spine	−0.078	0.444	0.057	0.475
Total hip	−0.190	0.060	0.029	0.715
Left femoral neck	−0.221	0.028	0.026	0.749
Right femoral neck	−0.160	0.114	0.002	0.980
Total body	−0.218	0.030	0.055	0.492
Nondominant ultradistal radius	−0.306	0.002	−0.013	0.872
**Correlation between PTH and BMD**				
Lumbar spine	−0.023	0.822	−0.022	0.788
Total hip	−0.011	0.911	−0.117	0.149
Left femoral neck	−0.087	0.391	−0.134	0.097
Right femoral neck	−0.077	0.449	−0.127	0.117
Total body	−0.023	0.825	−0.078	0.336
Nondominant ultradistal radius	0.114	0.259	−0.129	0.112

BMD: bone mineral density, 25(OH)D: 25-hydroxyvitamin D, 1,25(OH)_2_D: 1,25-dihydroxyvitamin D, PTH: parathyroid hormone, r: correlation coefficient.

## Discussion

The main findings in this study were that biochemical markers of bone turnover and metabolism, including vitamin D status, did not differ significantly between newly diagnosed CIS and MS patients with prevalent low bone mass and controls after adjusting for relevant confounders. There were no significant associations of biochemical parameters with bone status within or between the patient and control groups.

The lack of association between markers of bone turnover and BMD is in line with previous findings in ambulatory premenopausal female patients [22] and also in physically active patients of both sexes [23], both with more long-standing MS and higher EDSS than in our patients. Several studies of young MS patients (<55 years) with longer disease duration than our patients have found that vitamin D levels were not associated with reduced BMD [24]–[27]. Our findings are at odds with a study showing negative correlation between bALP and trochanter BMD [24]. Notably, these patients had much longer disease duration and more pronounced disability than our patients, and no other associations between biochemical bone markers and BMD were found. Nevertheless, one of these studies reported lower levels of the bone formation marker osteocalcin, higher bone resorption markers (pyridinoline and deoxypyridinoline), and higher PTH in premenopausal women with relapsing remitting MS compared to controls [22]. However, only PTH was inversely correlated with lumbar BMD [22]. Differences in disability levels, disease duration, biochemical parameters of bone metabolism, and measured BMD sites may contribute to the conflicting results.

Vitamin D deficiency may cause reduced acquisition of bone during growth and enhanced bone loss in adults [10], [14]. At latitudes ≥42° North (e.g. Oslo, Norway, 59° North) the solar UV-B radiation in winter is insufficient for cutaneous production of vitamin D [28]. The major circulating vitamin D metabolite, 25(OH)D, is sensitive to both recent UV exposure and vitamin D intake [10], [19]. Hydroxylation of 25(OH)D to bioactive 1,25(OH)_2_D by 25-hydroxyvitamin D-1α-hydroxylase (CYP27B1) in the kidneys is tightly regulated by Ca, phosphate, PTH, and by negative feedback from 1,25(OH)_2_D [10]. Both the genes encoding CYP27B1 and 25-hydroxyvitamin D-24-hydroxylase (CYP24A1) that catalyzes breakdown of 1,25(OH)_2_D may be associated with MS risk [29], [30]. PTH enhances the renal reabsorption of calcium and stimulates production of 1,25(OH)_2_D and bone resorption. 1,25(OH)_2_D increases intestinal absorption of Ca and phosphate. Vitamin D deficiency leads to decreased intestinal Ca absorption and secondary increased PTH [10]. Given the high prevalence of low BMD among the patients in our study, one might have expected to find perturbations in bone turnover and metabolism. However, the relationship between markers of bone formation (bALP) and resorption (NTX) and the correlations between biochemical indices of bone metabolism (25(OH)D and PTH, PTH and Ca) were consistent and not significantly different between patients and controls. Thus, the bone deficit found in these newly diagnosed patients seems less likely to be a result of a recent acceleration of bone turnover.

We did not find significant differences in measured 25(OH)D or 1,25(OH)_2_D, estimated vitamin D intake, or reported sun/UV exposure in patients compared to controls. This is at variance with studies that have reported lower 25(OH)D [25], [31]–[36] or 1,25(OH)_2_D [33] levels in MS patients than in controls, but concur with other studies showing no significant differences in 25(OH)D [37]–[40] or 1,25(OH)_2_D [37], [39], including patients with low disability [32] and short disease duration [31]. These differences might be due to small sample sizes and different selection of participants between studies (e.g. exclusion of subjects with possible negative bone-influential factors and controls chosen from hospital staff rather than general population). Higher EDSS has been found to be associated with reduced sun exposure [32]. As expected from the vitamin D measurements, our patients did not report different recent sun/UV exposure compared to controls. We confirm the positive association between serum 25(OH)D and 1,25(OH)_2_D previously reported in MS patients [37], [41], indicating dependency on the availability of 25(OH)D for 1,25(OH)_2_D synthesis, although the renal activity of CYP27B1 is strictly regulated.

Smoking is another potential risk factor for both MS and osteoporosis [14], [42] and was more common among patients than controls in our study. However, the proportion of current smokers was relatively low, and smoking did not influence the levels of NTX. Short-term glucocorticoid treatment of MS relapses can trigger immediate changes in bone metabolism and turnover [43], [44], but has not been consistently related to markers of bone turnover in MS [22]. The use of systemic glucocorticoids did not significantly influence the levels of NTX or bALP in our participants. Our findings cohere with observations in non MS-populations [45]. As the use of systemic glucocorticoids was mainly in the patient group, it could be argued that such treatment was approaching a status as an intermediary variable/mediator (in the causal path between MS and bone status) and should consequently not be adjusted for. When excluding the use of systemic glucocorticoids from the analyses of NTX, the difference in mean NTX between MS patients and controls remained non-significant (data not shown). However, both patients and controls could have and had used systemic glucocorticoids, and therefore this use was adjusted for.

The major strength of this study is the population-based design with rigorous recruitment of newly diagnosed incident cases. The public health care system in Norway provides equal free-of-charge access to medical services for all Norwegian citizens. It is therefore likely that almost all incident cases of MS and CIS in Oslo were invited to participate, and that the included patients were representative of the source population. The case-control design allowed comprehensive collection of relevant lifestyle factors as well as biological data, making it possible to adjust for potential confounders. Biochemical measures and BMD obviously complement each other, as do markers of bone turnover and metabolism, because turnover markers reflect but do not regulate bone metabolism. In addition, neither self-reported recent UV exposures, assessed intake of vitamin D, nor measured serum 25(OH)D differed significantly between patients and controls. Because serum 25(OH)D is a marker of recent sun exposure and vitamin D intake, this consistency is reassuring.

There are also several limitations to this study. Markers of bone turnover are affected by a number of factors [17], and even if we have taken many of these into account, we lacked data on genetics, gonadal steroids, and inflammatory cytokines. Because of the case-control design, there could be potential recall bias for lifestyle factors that were assessed by questionnaire. In order to minimize recall bias, the participants did not know their vitamin D or bone status when completing the questionnaire, which contained a wide range of questions regarding the diet, health, and lifestyle without giving prime focus to any section. Notably, at the time of examination there was not yet general awareness among patients or controls in Norway about of the possible role of vitamin D and bone health in MS. Although we aimed to minimize the impact of self-selection by randomly recruiting controls from the general population and by letting the patients recruit additional controls, we cannot rule out the possibility that persons with an unhealthy lifestyle participated to a lesser extent than persons with a healthier lifestyle. Smoking habits could be an indicator of such bias. Data from Statistics Norway (www.ssb.no\english) show that 17% of the Norwegian population aged 16–74 years and even fewer under 25 years were daily smokers in 2011. Thus, the smoking habits of our controls seem to be in line with the general population and do not suggest a bias towards particularly health-conscious controls. Moreover, body mass index (BMI), which is an indicator of general health and also associated with BMD and vitamin D status [46], did not differ between patients and controls [15]. BMI was also consistent with previous Oslo health surveys [47]–[49]. One of these evaluated the impact of self-selection and non-attendance on BMI and smoking and found that prevalence estimates were robust and that self-selection had little impact [50]. Importantly, most of the source population and the participating controls were likely too young to be concerned about osteoporosis, and low bone mass is asymptomatic. Knowledge of risk factors for osteoporosis is not evident, and in order to minimize differential selection bias we did not inform about such risk factors in the invitation letter.

Our previous study showed low bone mass at isolated skeletal sites but no significant difference in the total body bone mass in patients compared to controls [15]. Biochemical markers reflect the metabolism of the entire skeleton and not specific skeletal sites. We can therefore not completely exclude the possibility of a minor increase in bone turnover contributing over time, as increased turnover at some sites may have been insufficient to induce a difference in the systemic levels of the measured markers.

The low bone mass in these newly diagnosed patients with CIS and MS compared to controls was not mirrored by significant differences in bone turnover or metabolism. In addition, the lack of association between biochemical indices of bone metabolism and bone status might indicate a longstanding BMD deficit rather than a recent bone loss. Shared etiological factors between MS and osteoporosis such as vitamin D deficiency could cause low peak bone mass and low BMD at disease onset. The lack of difference in vitamin D status between patients and controls in our study does not rule out the possible role of low vitamin D in the pathogenesis of MS, as vitamin D measures after disease onset are not considered representative of vitamin D status prior to disease onset [51]. Alterations in behavior, medication, and the disease process itself may affect vitamin D status after disease onset. Risk factors may also normalize from susceptibility periods in early life or youth until the time in adult life when symptoms appear. Thus, obese female adolescents were found to have increased MS risk, but there was no association between adult body size and MS risk [52]. The findings presented here and previously [15] are therefore in line with the hypothesis that if vitamin D status exerts a major effect on MS risk, skeletal consequences of hypovitaminosis D should be apparent from the onset of disease. This suggestion does not exclude other possible links not related to vitamin D between MS and osteoporosis, including shared genetic risk factors, and genetic variation in the interleukin-6 gene is one example that has been associated with both diseases [53], [54].

We know that low bone mass may occur early in MS and that patients with MS are at high risk of osteoporosis and fractures. Newly diagnosed patients have many years of disease ahead. Osteoporosis, fractures, and their sequelae may therefore have important impact on the patients’ quality of life. Bone health in the early stage of MS deserves awareness and is far from fully explored. This lack of knowledge should trigger prospective studies on the mechanisms involved as well as prophylaxis and treatment.
